# Innovative Visualization and Quantification of Extracellular Vesicles Interaction with and Incorporation in Target Cells in 3D Microenvironments

**DOI:** 10.3390/cells9051180

**Published:** 2020-05-09

**Authors:** Enrico Ragni, Silvia Palombella, Silvia Lopa, Giuseppe Talò, Carlotta Perucca Orfei, Paola De Luca, Matteo Moretti, Laura de Girolamo

**Affiliations:** 1 IRCCS Istituto Ortopedico Galeazzi, Laboratorio di Biotecnologie Applicate all’Ortopedia, Via R. Galeazzi 4, I-20161 Milano, Italy; enrico.ragni@grupposandonato.it (E.R.); carlotta.perucca@grupposandonato.it (C.P.O.); deluca.paola@grupposandonato.it (P.D.L.); 2 IRCCS Istituto Ortopedico Galeazzi, Cell and Tissue Engineering Laboratory, Via R. Galeazzi 4, I-20161 Milano, Italy; silvia.palombella@grupposandonato.it (S.P.); silvia.lopa@grupposandonato.it (S.L.); giuseppe.talo@grupposandonato.it (G.T.); 3 Regenerative Medicine Technologies Laboratory, Ente Ospedaliero Cantonale (EOC), 6900 Lugano, Switzerland

**Keywords:** mesenchymal stem cells, extracellular vesicles, orthopedics, osteoarthritis, extracellular matrix, flow cytometry, confocal microscopy, microfluidics

## Abstract

Extracellular vesicles (EVs) showed therapeutic properties in several applications, many in regenerative medicine. A clear example is in the treatment of osteoarthritis (OA), where adipose-derived mesenchymal stem cells (ASCs)-EVs were able to promote regeneration and reduce inflammation in both synovia and cartilage. A still obscure issue is the effective ability of EVs to be internalized by target cells, rather than simply bound to the extracellular matrix (ECM) or plasma membrane, since the current detection or imaging technologies cannot fully decipher it due to technical limitations. In the present study, human articular chondrocytes (ACHs) and fibroblast-like synoviocytes (FLSs) isolated from the same OA patients were cocultured in 2D as well as in 3D conditions with fluorescently labeled ASC-EVs, and analyzed by flow cytometry or confocal microscopy, respectively. In contrast with conventional 2D, in 3D cultures, confocal microscopy allowed a clear detection of the tridimensional morphology of the cells and thus an accurate discrimination of EV interaction with the external and/or internal cell environment. In both 2D and 3D conditions, FLSs were more efficient in interacting with ASC-EVs and 3D imaging demonstrated a faster uptake process. The removal of the hyaluronic acid component from the ECM of both cell types reduced their interaction with ASC-EVs only in the 2D system, showing that 2D and 3D conditions can yield different outcomes when investigating events where ECM plays a key role. These results indicate that studying EVs binding and uptake both in 2D and 3D guarantees a more precise and complementary characterization of the molecular mechanisms involved in the process. The implementation of this strategy can become a valuable tool not only for basic research, but also for release assays and potency prediction for clinical EV batches.

## 1. Introduction

Extracellular vesicles (EVs) are nanoparticles delimited by a lipid bilayer that are naturally released from almost all cell types [1]. Particles differ in both size and origin, either endosomal or plasma membrane-derived [2]. Endosomal EVs are generally smaller (50–150 nM) and referred to as exosomes, whereas plasma-membrane EVs are larger (>100 nM) and defined as microvesicles. The difference in their origin suggested a differential fingerprint, for both cargo and functions [2], although, to date, no molecular signatures have been defined to sharply distinguish these two categories of nanoparticles. Therefore, due to the absence of a consensus on specific markers for EV subtypes, they have recently been divided into small (<200 nM) and large (>200 nM) particles, superseding the classical exosome and microvesicle definition, with a more general definition based on either substantial overlap for some determinants in their surface biochemical composition (presence of CD63/CD81/CD9, etc.) or cell of origin [3]. In living organisms, EVs act as intercellular messengers to transfer biological signals such as nucleic acids, proteins and lipids [4]. Due to this function of cargo transfer, EVs have been recently studied as therapeutics for the delivery of both natural and engineered loads [5]. In this perspective, EVs, especially when collected from mesenchymal stem cells (MSCs), showed innate therapeutic properties in the frame of regenerative medicine [6]. Notably, pre-clinical studies shed light on the potential of MSC-EVs for several pathologies affecting the liver, heart, kidney, brain, bone, and joints [6]. In particular, in osteoarthritis (OA), characterized by both degenerative and inflammatory processes, MSC-EVs were able to promote the regeneration of joint components, such as synovium and cartilage, and to reduce pain [7] through the delivery of their cargo enriched in anti-inflammatory and trophic factors and miRNAs [8].

Mandatory to understanding and ameliorating EV for potential future clinical use is the correct visualization and quantification of their uptake at the cell, tissue, and organ levels in explorative and pre-clinical settings. At present, the majority of available data has been generated in vitro, although some major issues have still to be faced to consistently translate knowledge in clinics. In this frame, regardless of the protocol used, studies concerning EV internalization rely mainly on the detection of fluorescent signals in target cells or tissues incubated with pre-labeled EVs. Among the compounds for labeling EVs, membrane-specific fluorescent dyes (PKH67, PKH26, etc.) [9] or membrane-permeable carboxyfluorescein diacetate succimidyl ester (CFSE) [10] are the most used. Except for very expensive and rare high-resolution instrumentation, in the majority of laboratories, the available instrumentation allows fluorescence detection through conventional flow cytometry and/or fluorescent or confocal microscopy. In our experience, despite giving valuable information such as the ability of EVs from adipose-derived MSCs to interact with fibroblast-like synoviocytes (FLSs) isolated from OA patients [11], these techniques do not allow an exact understanding of the level of interaction between EVs and cells. This may be ascribed to the fact that recipient cells are subjected to trypsin digestion and multiple washing steps, possibly hampering EV-cell interaction and consequently the detection of the fluorescent signal [12]. Moreover, while by flow cytometry it is impossible to discern internalized EVs from those simply attached to the cell surface or bound to the plasma membrane, still this is very difficult also by confocal microscopy on standard monolayer cultures. This is due to the reduced z-axis thickness of target cells grown in an unnatural 2D environment on plastic cell culture surfaces, which makes it difficult to clearly discriminate between the extracellular matrix, plasma membrane, and cytoplasm [13]. 

Due to the above-mentioned reasons, there is an urgent need to establish advanced models to deeply characterize the interaction of EVs with single cells. In fact, the development of new tools able to discriminate and quantify EV adhesion vs internalization is mandatory to study molecular mechanisms in basic research and establish release assays to characterize alternative EV batches for regenerative medicine purposes. In this context, 3D culture models, like the ones that can be established in microfluidic devices, represent an invaluable tool. Culturing cells in a 3D environment that resembles their natural niche allows indeed to study their behavior in an in vivo-like milieu where cells may acquire a more natural and tridimensional morphology. In this scenario, confocal microscopy could make possible to discriminate between cells with internalized EVs and cells that have interacted only superficially with EVs, a crucial issue for biodistribution and potential enhancement of EV uptake beside simple interaction with target cells, for both basic research and clinical applications. Among the most diverse approaches to establish a 3D culture, microfluidics provides several advantages, such as the low number of samples required and the possibility to establish spatially confined cocultures, creating compartmentalized environments that can simulate human tissues, organs, or pathology features [14].

This work focused on the technical advantage of studying the interaction and internalization of MSC-derived EVs in donor-matched FLSs and articular chondrocytes (ACHs) isolated from OA patients in a 3D physiological-like microenvironment. 3D confocal microscopy data were compared with those obtained through conventional flow cytometry on cells grown in 2D on traditional plastic surfaces. Finally, the role of the hyaluronic acid (HA)-based matrix in EV docking was dissected to confirm HA as a crucial factor in the trafficking of EVs in target cells. We hypothesized that 2D and 3D approaches should be combined to achieve complementary data on cell interaction with EVs and that this strategy could represent a valuable tool for both basic types of research on EV-cell molecular mechanisms and clinical translation for therapeutic EV batches choice.

## 2. Materials and Methods

### 2.1. Ethics Statement

This work was completed at IRCCS Istituto Ortopedico Galeazzi. Institutional Review Board approval (San Raffaele Hospital Ethics Committee approval on date 8 March 2018, registered under number 6/int/2018) was obtained before the beginning of the study. Sampling was performed after the procurement of patient informed consent (CI_REGAIN_adulto_v2) and following the 1964 Helsinki declaration and its later amendments.

### 2.2. Adipose Tissue-Derived Mesenchymal Stem Cell (ASC) Isolation and Culture

Adipose waste material was collected from three female donors (54 ± 8 years old) undergoing liposuction. Tissue was processed as previously reported [11]. Briefly, after type I collagenase (Worthington Biochemical Co., Lakewood, NJ, USA) digestion at 37 °C for 30 min, tissues were filtered (100 μM cell strainer) and centrifuged at 1000× *g* (5 min, RT). Pellets were suspended in DMEM + 10% FBS and seeded at 5 × 10^3^ cells/cm^2^ (37 °C, 5% CO_2_, 95% humidity).

### 2.3. ASC Characterization by Flow Cytometry

ASCs at passage three were analyzed by flow cytometry with a CytoFLEX flow cytometer (Beckman Coulter, Fullerton, CA, USA), collecting at least 10,000 events. Antibodies used to confirm ASC phenotype [15] were: anti-CD44-PE (Cat# 130-110-293), CD90-FITC (clone REA897), CD105-PerCP-Vio700 (clone REA794), CD45-PE Vio770 (clone REA747) (Miltenyi Biotec, Bergisch Gladbach, Germany). Doublets were removed from analysis gating events on FSC-H and FSC-A plot.

### 2.4. EV Production

ASCs at passage three and 90% confluence were washed twice with PBS, and DMEM without FBS was added. After 48 h, supernatants were collected and serially centrifuged at 376× *g*, 1000 × *g*, 2000 × *g* and twice at 4000× *g* to remove floating cells and debris. When fluorescent EVs were needed, the supernatant was labeled with 10 μM CFSE (Sigma-Aldrich, Milan, Italy) for 1 h at 37 °C. Eventually, EVs or CFSE-labeled EVs were collected by ultracentrifugation at 100,000× *g* for 3 h at 4 °C. No more than 25 μL supernatant were left and pellets were washed with 25 mL PBS to remove excess dye and final pellets, again with no more than 25 μL supernatant, were suspended in PBS, 100 μL per 25 mL of initial culture supernatant. Initial CFSE concentration at this step was 1:4000 reduced. EVs from the three ASCs isolates were pooled for incorporation studies. To confirm a lack of major protein contamination, the number of particles was related to total protein amount and EV batches considered of good purity when falling in the 10^8^ to 10^10^ particle/μg protein range, as described in [16].

### 2.5. EV Characterization by Flow Cytometry

CFSE-EVs were analyzed by flow cytometry with a CytoFLEX flow cytometer calibrated with FITC-fluorescent microbeads to allow the detection of fluorescent particles as small as 100 nM, as previously reported [11]. Calibration standards were 160, 200, 240, and 500 nM sizes (Biocytex, Marseille, France). EVs were 1:10,000 diluted in PBS and 100 µL stained with anti-CD63-APC (clone H5C-6) and CD81-APC (clone 5A6) (Biolegend, San Diego, CA, USA) antibodies for 30 min at 4 °C. Gains were: FSC = 106, SSC = 61, FITC = 272, PE = 116, and PC7 = 371. After incubation, samples were diluted with PBS to 1000 μL before analysis. At least 10,000 events were collected. CFSE-EVs were first compared in the FITC channel with a PBS+CFSE sample used as background signal, to gate only stained EVs. Only events falling in this gate were used to analyze Ab unstained and stained CFSE-EVs and cytograms in the APC channel overlaid to detected positive particles. This strategy of initial FITC gating allowed to remove from the analysis Ab aggregates that, being APC labeled, do not give autofluorescence spillover in the FITC channel.

### 2.6. EV Characterization by Transmission Electron Microscopy

Purified EVs were blotted on Formvar carbon-coated grids for 10 min and drops removed. After negative stain (2% uranyl acetate aqueous suspension, 10 min), the grid was dried at RT. TALOS L120C transmission electron microscope (Thermo Fisher Scientific, Waltham, MA, USA) at 120 kV was used to examine the samples.

### 2.7. EV Characterization by Nanoparticle Tracking Analysis (NTA)

Nanosight LM10-HS system (NanoSight Ltd., Amesbury, UK) was used to visualize purified EVs that were 1:100 diluted in PBS. Three 30 s recordings were performed for each sample. Dedicated software provided both the concentration measurements and the high-resolution particle size distribution profiles.

### 2.8. ACHs and FLSs Isolation and Culture

Donor-matched FLSs and ACHs were isolated from synovial membranes and full-thickness cartilage explants obtained from three male donors (67 ± 5 yo) undergoing hip replacement. Synovial membranes were treated as previously described [11]. Briefly, minced tissues were enzymatically digested (37  °C, 3 h) by 0.25% w/v type I collagenase (Worthington Biochemical Co., Freehold, NJ, USA). Digested samples were filtered through a 100 μM cell strainer and centrifuged (376× *g*, 5 min). Pellets were suspended in DMEM supplemented with 10% FBS and FLSs selected for plastic adherence. Cartilage explants were processed as previously described [17,18]. Briefly, minced tissues from the femoral head were incubated overnight at 37 °C with 0.15% w/v type II collagenase (Worthington Biochemical Corporation). After centrifugation, cells were seeded in DMEM supplemented with 10% FBS, and ACHs selected for plastic adherence. Both ACHs and FLSs were maintained in an incubator at 37  °C in a humidified atmosphere with 5% CO_2_ and used for the following experiments at the same passage for donor-matched cells.

### 2.9. Determination of EV Interaction with ACHs and FLSs in 2D Cultures by Flow Cytometry

ACHs and FLSs were seeded in duplicate each at 10,000 cells/cm^2^ in 24-well plates. After 24 h (time 0 for interaction assessment), the medium was removed and replaced with either DMEM + 10% FBS (ultracentrifuged for 24 h at 100,000× *g* to remove endogenous EVs), or DMEM + 10% FBS + CFSE-labeled EVs (ratio of 50,000 EVs per seeded cell). Due to average 1:50 purified CFSE-EVs dilution in culture medium, residual CFSE concentration during EVs uptake was below 0.1 nM, greatly inferior to the recommended concentration range (0.1–10 μM) for labeling and therefore avoiding a specific and EV-independent cell staining. After 1, 6, and 24 h, the medium was removed and cells detached to be compared with cells detached at time 0 before EV supplementation. ACHs and FLSs were analyzed with a CytoFLEX flow cytometer and fluorescence in the FITC channel detected. To adjust for technical differences in the number of seeded cells or cell growth between seeding and flow cytometer analysis leading to slightly different EVs to cells ratios, the fluorescence values were normalized per counted events in the cell gate under SSC-A vs FSC-A under the assumption of a linear and inverse proportion between fluorescence intensity and EVs to cell ratio (e.g., double cell number leading to half mean fluorescence intensity and vice versa). The assumption is based on the working conditions that are far below the saturation EVs to cells ratio (100,000 to 1) previously demonstrated in [11].

### 2.10. Hyaluronic Acid (HA) Matrix Removal and Determination of EV Interaction in 2D Cultures by Flow Cytometry

To remove HA matrix before seeding in 24-well plates, after trypsin detachment, both ACHs and FLSs were split in two, and one aliquot was treated for 10 min at 37 °C with 10 U/mL hyaluronidase (Sigma-Aldrich), whereas the other aliquot was incubated for the same time without the enzyme (mock). After two washes, treated cells were supplemented with 1 mM 4-methylumbelliferone (4-MU) (Sigma-Aldrich), an HA synthesis inhibitor, in the complete medium, and eventually, both hyaluronidase + 4-MU treated and untreated cells were seeded as previously described. 4-MU was left all night in treated cells. The following day, CFSE-labeled EVs (ratio of 50,000 EVs per seeded cell) were added in both treated and untreated cells and 4-MU was maintained in treated cells. Flow cytometry was performed at 0 (before EV addition) and 24 h and 4-MU treated (±EVs) *vs* untreated (±EVs) samples compared. Fluorescence values were normalized by detected events under SSC-A vs FSC-A plot.

### 2.11. Assessment of CFSE-Labeled EV Transfer to ACHs and FLSs by Microscopy

As per paragraph 2.9., ACHs and FLSs were seeded in 8-wells multi-chamber glass slides and CFSE-labeled EVs (ratio of 50,000 EVs per seeded cell) were supplemented the day after. Before seeding, both cell types were labeled with CellTracker™ Deep Red Dye (Invitrogen, Eugene, OR, USA) following the manufacturer’s instructions. Briefly, after detachment, 150,000 cells were suspended in serum-free medium and the dye solution was added at a final concentration of 500 nM. Cells were then incubated for 30 min and washed once before being counted and seeded. Images were taken at 0 and 24 h after EV addition.

### 2.12. Design and Fabrication of the Microfluidic Chip

**Figure 1:**
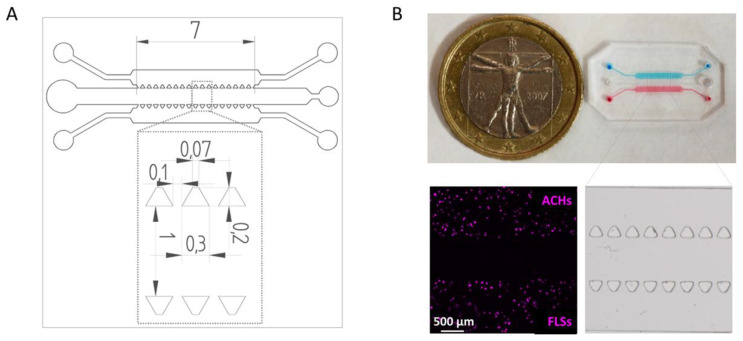
Design and fabrication of the microfluidic device. (**A**) Technical drawing of the microfluidic device with dimensions of trapezoidal posts and channels expressed in mm. (**B**) Top-view of the microfluidic device with ACH (blue) and FLS (red) compartments. The inset shows a stereomicroscope image of the microfluidic chip features. Representative picture showing ACHs and FLSs embedded in fibrin gels after staining.

The microfluidic chip was designed using Solid Edge ST9 CAD Software (Siemens AG, Texas, USA). The device comprised two gel compartments to inject fibrin-embedded tissue-specific cells, which are ACHs and FLSs, divided by a central channel for the injection of medium and EVs. The width and the height of the channels were 1 mM and 150 µM, respectively, for a total length of 7 mM. The three channels were separated by regularly spaced trapezoidal posts (basis: 0.07 mm/0.3 mm; height: 0.2 mM; distance: 0.1 mM) (Figure 1).

The mold for the microfluidic chip was generated in acrylic resin using a DLP 3D Printer (ASIGA, Sydney, Australia). Microfluidic chips were then produced by replica molding using PDMS SYLGARD^®^ 182 Silicone Elastomer (Dow Corning, Michigan, USA), poured on 3D printed molds and cured at 65 °C for 120 min in oven, finally punched to obtain the ports (gel compartments: 1 mM Ø for inlet/outlet; central channel: 1 mM Ø for inlet, 2 mM Ø for outlet) and bonded to a glass slide through plasma bonding using a Plasma Cleaner (Harrick Plasma Cleaners, New York, USA).

### 2.13. Cell Culture in the Microfluidic Chip

Before seeding in the microfluidic device, ACHs and FLSs were stained with CellTracker™ Deep Red Dye (Invitrogen, Eugene, Oregon, USA). For HA removal experiments, cells were treated with hyaluronidase before staining, as previously described. For fibrin gel embedding, cells were suspended at 3 × 10^6^ cells/mL in human thrombin (4 UI/mL diluted in 40 mM CaCl_2_, Tisseel kit, Baxter), mixed 1:1 with human fibrinogen (20 mg/mL diluted in PBS, Sigma-Aldrich), and injected in the respective microfluidic compartments. The chips were incubated at RT for 7 min to let the gels polymerize. This procedure resulted in a final cell concentration of 1.5 × 10^6^ cells/mL in 10 mg/mL fibrin gels. 

Complete medium with or without 4-MU depending on the experimental groups was injected in the central channel of the chip after gel polymerization. After culturing chips for 24 h at 37 °C and 5% CO_2_, 50,000 EVs per cell were injected in the central channel with or without 4-MU depending on the experimental setting. Chips were fixed at the desired time points (1, 6, and 24 h after EV injection) with 2% paraformaldehyde for 15 min at RT. Chips not injected with EVs were also fixed and used to define background fluorescence signals. Pictures were taken by a confocal microscope (Leica SP8) acquiring a z-stack of 150 µM corresponding to the entire channel height. Each sliced picture was taken at a distance of 1 µM.

### 2.14. Quantification of Internalized EVs

To evaluate the interaction between cells and EVs, image analysis was performed by ImageJ software. We developed a macro script to automatize the analysis process using as input two-channel z-stack images acquired by confocal microscopy. For each stack image, the macro performed four counting steps: 1. count of the total number of cells and generate ROIs of the cell areas, 2. count of the number of cells with internalized EVs, 3. count of the number of cells interacting with EVs (either on the surface or internalized), 4. evaluation of the signal intensity (gray value) of the EV channel, using the cell ROIs generated in step 1. In particular, for the first phase, a “Z project” of the maximum signal intensity was generated from the cell channel and the background was removed using the command “Subtract Background”. After applying the “Watershed” filter to separate close cells, the “Analyze particles” tool (minimum cell size: 80 µm^2^) was used to count the total number of cells. The gray value for the signal of the cell tracker was 8 bit sampled corresponding to 255 values of gray intensity. The threshold was set to include as much signal as possible (from 15 to 255), after subtracting the dark background, to include the whole cell. The selected threshold was the result of an empirical iterative process, where different independent expert biologists analyzed sample images in order to optimize the value. For the second phase, the ImageJ plugin “Colocalization” (https://imagej.nih.gov/ij/plugins/colocalization.html), was used to generate an image of the colocalized points, representing the EVs internalized by the cells. On this image, the “Analyze particles” tool was used to determine the number of cells with internalized EVs, considering positive only the cells that colocalized with EV signal with a minimum size of 5 µM^2^. Subsequently, the function “AND” between the stack channel of cells and the stack channel of EVs was used to evaluate the presence of close signals in both channels. Boolean “AND” function is similar to Colocalization but less restrictive allowing to include also the cell with EVs on the membrane. The resulting image, after applying the “Z project” command, was examined with the “Analyze Particles” tool with a threshold size of 64 µM^2^ (80% on the minimum cell size) determining the number of cells interacting with the EVs, regardless if present on the surface and/or internalized. Furthermore, the “Measure” tool was used to evaluate the EV signal strength in the ROIs generated in the first step. This section has been performed in the Z Project of the EV channel to evaluate all the EVs near or inside the cells. After that, a new stack was obtained subtracting (Image Calculator, Subtract) to EVs channel stack the colocalized point stack, to evaluate only the signal strength only on the cell surface. Also, in this case, the “Measure“ command was used on the Z Project of the resulting stack using the same ROIs generated in step 1.

### 2.15. Statistics

Statistical analysis was performed using GraphPad Prism Software version 5 (GraphPad, San Diego, CA, US). Normal data distribution was assessed by the Kolmogorov–Smirnov normality test. Student’s *t*-test was used to compare data. Level of significance was set at *p*-value  <  0.05.

## 3. Results

### 3.1. ASCs and ASC-EVs Showed Typical Features Confirming Their Identity

**Figure 2:**
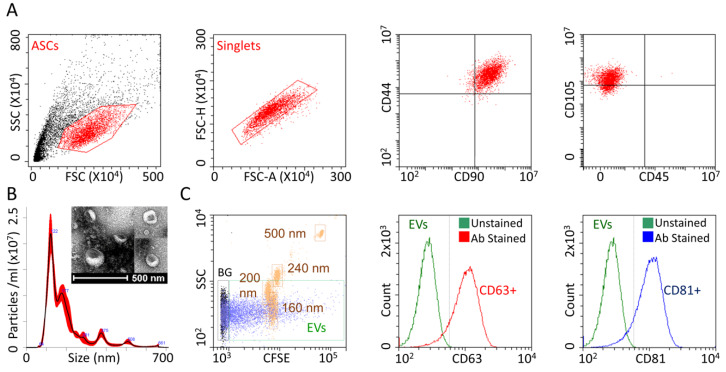
ASC and ASC-EV phenotype. (**A**) After FSC/SSC gating and singlets definition, ASCs resulted positive for CD90/44/105 MSC markers and negative for CD45 determinant. Representative cytograms of a single population are shown. (**B**) The size distribution of nanoparticles by NanoSight particle-tracking analysis and transmission electron micrographs of ASC-EVs showing particles with characteristic cup-shaped morphology (**C**) The first panel shows the merged cytograms of FITC-fluorescent nanometric beads (160 nM, 200 nM, 240 nM and 500 nM, squared in brown) used for flow cytometer calibration and CFSE-fluorescently labeled ASC-EVs (total population in violet, CFSE positive events squared in green as EVs), both gated with respect to PBS+CFSE background (total population in black and squared in black as BG), and confirmed instrument sensitivity to identify ASC-EVs as small as below 200 nm. Events gated in the EVs trace were used for assessing CD63/81 staining shown in the last two panels made by merging unstained and Ab stained (CD63 in red and CD81 in blue) CFSE-EVs cytograms. Both EV markers were detected on the majority of isolated ASC-EVs. One representative donor is shown.

ASCs homogeneously expressed mesenchymal antigens CD44, CD90 and CD105, being negative for hematopoietic marker CD45 (Figure 2A), confirming their identity. As revealed by NTA, purified ASC-EVs mostly ranged between 50 nM and 400 nM (Figure 2B), with the majority of particles resulting in smaller than 200 nM. EV size was further confirmed by transmission electron microscopy (Figure 2B). A calibrated flow cytometer able to identify particles as small as 100 nM [8,11] confirmed the NTA dimensional range (Figure 2C). The absence of EVs major protein contamination, that can influence size determination with particle aggregates, was confirmed by protein analysis of EV batches that resulted to have 0.33 × 10^9^ ± 0.12 particles/μg protein (N = 4, mean ± SEM), falling in the 10^8^ to 10^10^ range claimed for pure preparations [16]. Flow cytometry also confirmed the solid (>90%) presence of the EV markers CD63 and CD81 (Figure 2C).

### 3.2. 2D Experiments Showed a Higher Interaction of ASC-EVs with FLSs Than ACHs

**Figure 3:**
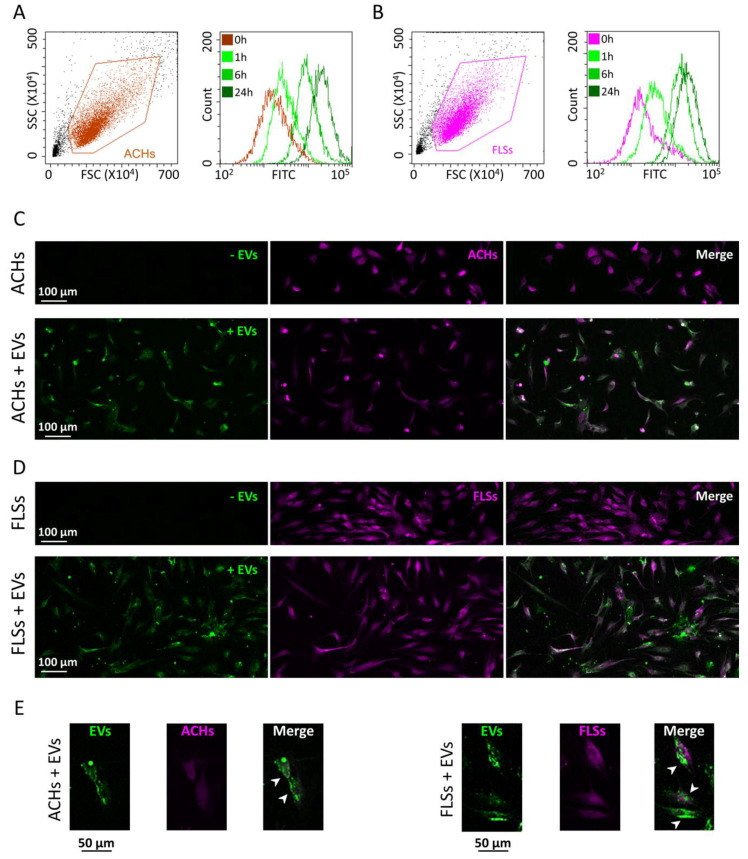
ASC-EV interaction with ACHs and FLSs over time in 2D. (**A**,**B**) Cytograms at time 0, 1-6-24 h for ACHs and FLSs incubated with ASC-EVs showing an increase in FITC-channel intensity signal over time. One representative donor is shown. (**C**,**D**) Images at time 0 and after 24 h of ACHs and FLSs grown in 2D and incubated with ASC-EVs. EVs were CFSE-stained and are shown in green, whereas cells were stained with CellTracker™ Deep Red Dye and are shown in violet. One representative donor is shown. (**E**) Magnification of ACHs and FLSs interaction with ASC-EVs that clearly stain in a punctuate fashion the cytoplasm and perinuclear area of single cells, as indicated in the merge pictures by the white arrows.

**Figure 4:**
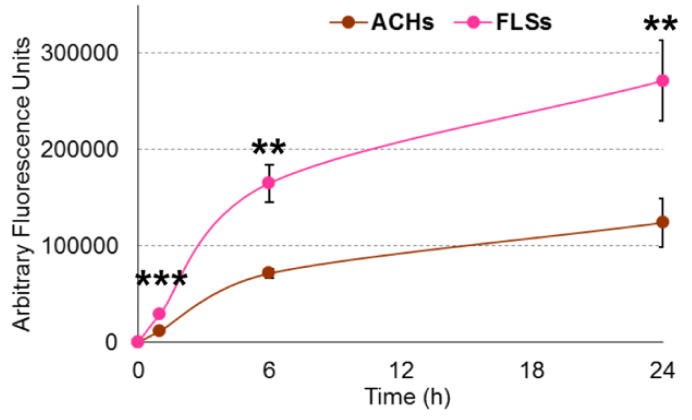
Differential ASC-EV interaction with ACHs and FLSs over time in 2D. Fluorescence intensities in the FITC channel for ACHs and FLSs over time. N = 3, values are shown as mean ± SE Significance for *p*-value < 0.05. ** for *p*-value < 0.01 and *** for *p*-value < 0.001.

ACHs and FLSs grown on 2D surface were incubated for 24 h with CFSE-labeled EVs, and interaction kinetics scored at 1, 6, and 24 h. Flow cytometry demonstrated the fluorescence gain over time in both cell lines. At 24 h, both ACHs and FLSs resulted to be CFSE positive, with a complete shift of the fluorescence peak (Figure 3A,B). EVs: ACHs of 50,000:1 did not lead to uptake saturation, since halving the cell number, and therefore doubling the available EVs per cells, resulted in a two-fold increase in detected fluorescence (data not shown). Fluorescence microscopy further supported the detection of EV interaction with the whole cell population (Figure 3C,D), with particles and particle clusters in the cytoplasm (Figure 3E) indicating a specific and EV-related process rather than unspecific labeling, given by free or protein aggregate-bound and released CFSE remnant, that typically results in a diffuse staining. FLSs did not show a linear time-dependent gain of signal, due to a flection in the fluorescence increment curve between 6 and 24 h. The same trend was observed also in ACHs, suggesting a conserved mechanism (Figure 4). Eventually, comparing flow cytometry fluorescence plots, the interaction between EVs and FLSs resulted statistically higher than with ACHs at all time points (Figure 4).

### 3.3. 3D Culture Imaging Allowed to Discriminate Internalized ASC-EVs Both in ACHs and FLSs

**Figure 5:**
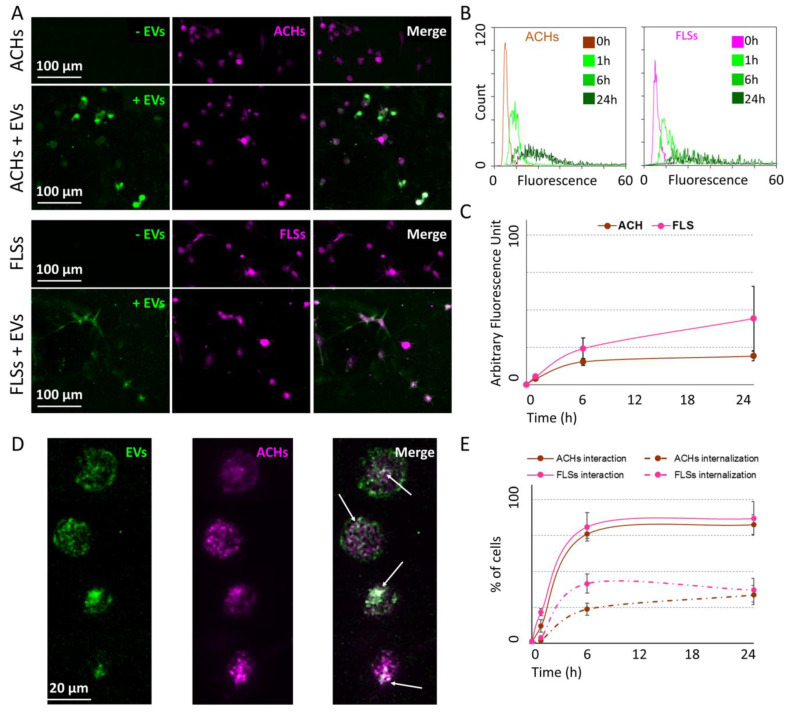
ASC-EV interaction with and incorporation in ACHs and FLSs over time in 3D microfluidic devices. (**A**) Pictures at time 0 and after 24 h of ACHs and FLSs grown in 3D chips and incubated with ASC-EVs. EVs were CFSE-stained and are shown in green, whereas cells were stained with CellTracker™ Deep Red Dye and are shown in violet. The merged pictures clearly show the overlapping of the signal of ASC-EVs with cells. One representative donor is shown. (**B**) Green fluorescence intensity at different time points of ACHs and FLSs incubated or not with ASC-EVs inside the 3D microfluidic device. (**C**) The trend over time of mean green fluorescence of ACHs and FLSs incubated with ASC-EVs. N = 3, values are shown as mean ± SE. Significance for *p*-value < 0.05. (**D**) Representative pictures of ASC-EVs (green) incubated with ACHs (violet) after 24 h in 3D microfluidic device. EVs clearly surrounded ACHs and were also internalized, as indicated in the merge pictures by the white arrows. (**E**) Trend over time of the number of cells either interacting with ASC-EVs regardless outside or inside (continuous line) or with internalization (dashed line). N = 3, values are shown as mean ± SE. Significance for *p*-value < 0.05.

After being embedded in 3D fibrin gel, ACHs and FLSs were incubated with EVs and interaction at different time points was detected by confocal microscopy. Similar to 2D cultures, pictures of 3D cultures confirmed that after 24 h EV fluorescence was clearly associated with both ACHs and FLSs, including cell prolongations (Figure 5A). The analysis of the intensity of total EV fluorescence, regardless of its cell location, demonstrated an increase over time for both ACHs and FLSs. Differently from monolayer cultures where the fluorescence peaks were compact at all time points, in 3D cultures at 6 and 24 h the fluorescence intensity spectra were more distributed (Figure 5B). Supporting 2D data, the saturation mechanism and the higher EV interaction with FLSs compared to ACHs were confirmed, although not reaching statistical significance (Figure 5C). A deeper examination of the images allowed us to discriminate cells with internalized particles (Figure 5D), thus providing more information than classical 2D cultures. As reported in Figure 5E, the number of interacting (inside + outside EVs) ACHs and FLSs increased over time, reaching a plateau phase (around 80% in both cell types) 6 h after injection. Notably, although EVs started to interact at 1 h, incorporation was not detected at this time point. At 6 h, 24% and 42% of ACHs and FLSs resulted in CFSE positive inside their cytoplasm, respectively. As per total fluorescence and total interaction, the incorporation at 24 h reached a plateau for both ACHs (34% of cells with internal fluorescence) and FLSs (37%).

### 3.4. Hyaluronic Acid Removal Affected ASC-EV Interaction with Cells in 2D but Not in 3D Cultures

**Figure 6:**
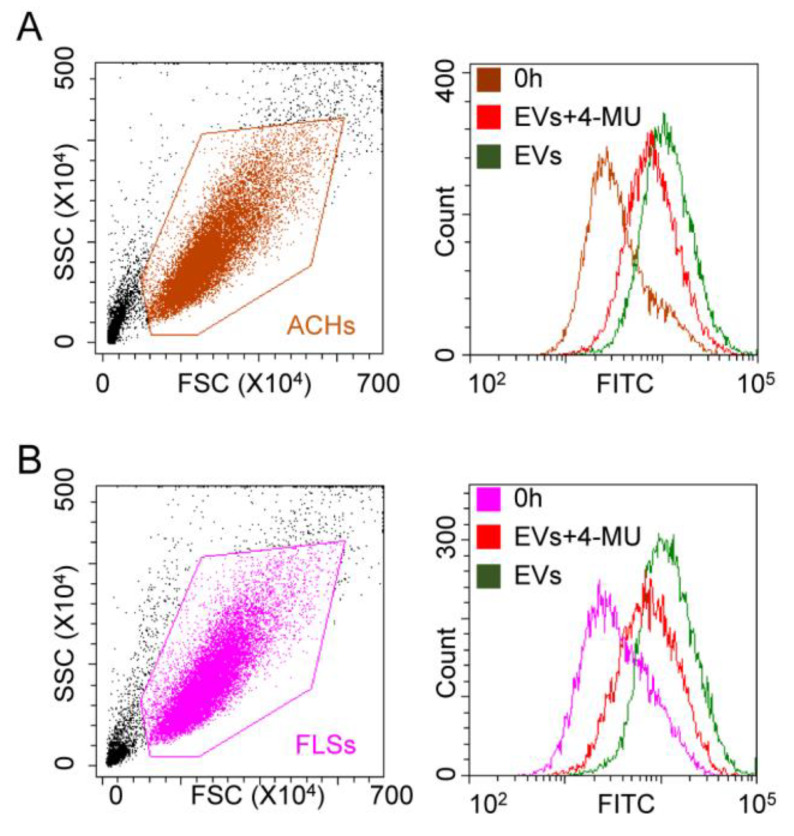
HA removal effect on ASC-EV interaction with ACHs and FLSs in 2D. (**A**,**B**) Cytograms showing fluorescence at time 0 and after 24 h for both ACHs and FLSs incubated with ASC-EVs, in presence and absence of HA-synthesis inhibitor 4-MU. HA removal reduces fluorescence increase at 24 h. 4-MU treated samples at time 0 are not shown for clarity, since no differences with control samples at time 0 emerged. One representative donor is shown.

Cells treated with hyaluronidase and maintained in complete medium containing hyaluronan synthesis inhibitor 4-methylumbelliferone (4-MU) were co-incubated with EVs. Fluorescence was detected at 24 h with either flow cytometry for 2D cultures or confocal microscopy for 3D microfluidic devices. Flow cytometry confirmed the significant (*p*-value < 0.05) reduction (42 ± 7%) in fluorescence in FLSs (Figure 6A). The diminution was also observed in ACHs with a similar percentage (47 ± 1%) (Figure 6B).

**Figure 7:**
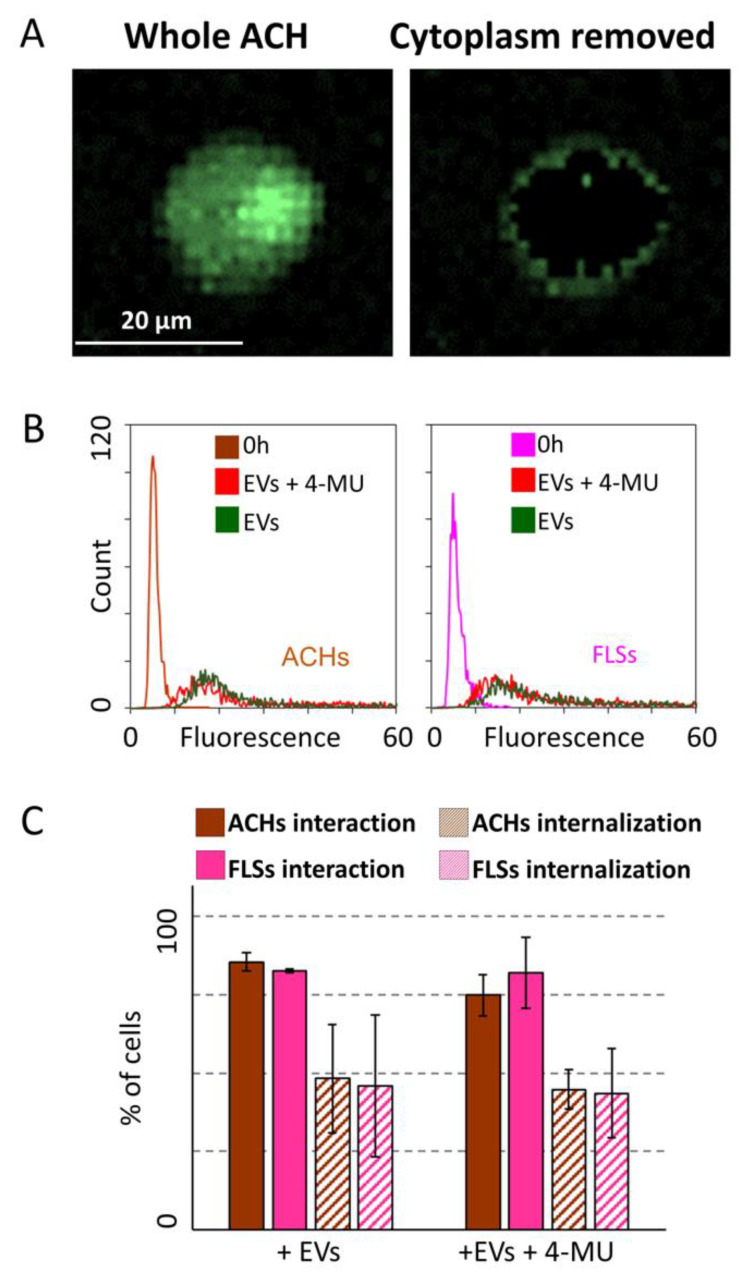
HA removal effect on ASC-EV interaction with and incorporation in ACHs and FLSs in 3D microfluidic devices. (**A**) Image of a selected ACHs before and after removal of fluorescence signals associated with the cytoplasm. The presence of EVs outside the cell body is clear. (**B**) EV fluorescence intensity at time 0 and 24 h of ACHs and FLSs treated or not with 4-MU inside the 3D microfluidic device. 4-MU treated samples at time 0 are not shown for clarity, since no differences with control samples at time 0 emerged. N = 3 (**C**) ASC-EVs interacting with or internalized in ACHs or FLSs with or without treatment with 4-MU at 24 h. N = 3, values are shown as mean ± SE. Significance for *p*-value < 0.05.

When analyzed in the 3D-based model, both ACHs and FLSs treated with 4-MU showed the presence of a fluorescent signal outside the cell membrane (Figure 7A and Supplementary File 1). The analysis of signal intensity did not result in decreased interaction between ASC-EVs and cells compared to untreated cells (Figure 7B). Consistently, 4-MU treatment did not alter the percentage of both ACHs and SFLs interacting with or incorporating EVs (Figure 7C).

## 4. Discussion

In this work, classical flow cytometry on 2D cultures and confocal microscopy for 3D cultures were used to identify and dissect the interactions between ASC-EVs and ACHs and/or FLSs by combining the possible complementary information given by these two different systems. Before performing any experiment, the identity of ASCs was confirmed by assessing the expression of standard surface markers [15] and ASC-EVs were characterized obtaining data consistent with the definition of “small EVs” that was recently released by the International Society for Extracellular Vesicles, in place of the old term “exosomes” [3]. For the first time, a clear distinction between adhesion of ASC-EVs on the cell surface and cell uptake was demonstrated through the use of a 3D microfluidic device. Moreover, the role of the extracellular matrix, and in particular of HA, in implementing EV docking was taken into account. The identification of a combined strategy of investigation would indeed be useful for basic research as well as for release assays and potency prediction for clinical EV batches.

In the last years, EV biogenesis started to be deciphered by a large body of evidence [3]. On the contrary, our understanding of the EV-cell interaction mode is in its infancy. This is a crucial key point since EVs, to exert their function, must firstly interact with recipient cells, being known that a preferential bind to specific target cell types occurs in vivo and, presumably, in humans [5]. In several in vivo studies that systemically administered EVs of different origins, a large proportion of injected particles accumulated in the spleen and liver [19,20,21]. Therefore, the biodistribution issue emerged as a pivotal aspect for safety and efficacy of EVs, to successfully improve targeted EV delivery. Together with internalization and cargo release, the ability of EVs to bind specific cell types could be used and modified to produce more efficient EVs, such as engineered drug delivery platforms to desired sites of action. In order to investigate and shed light on this issue, a profound knowledge of the molecular mechanisms behind EV-cell binding is mandatory, together with the development of dedicated and advanced technical platforms for detection and analysis.

To date, several molecules have been postulated to direct EV-cell recognition, like lipids, glycans, laminin or fibronectin via integrins [22,23,24,25]. These results were mainly obtained through conventional detection techniques that are currently used to quantitate EVs and visualize their uptake, including flow cytometers and confocal microscopes. Although useful milestones and mandatory for analysis, the aforementioned tools have some limitations. Flow cytometry can detect the fluorescence increase in target cells when incubated with labeled EVs. In this work, by flow cytometry, FLSs grown on 2D surfaces confirmed the previously observed time-dependent increase in fluorescence together with the saturation over time [11]. Similar kinetics was scored for donor-matched ACHs, confirming a conserved EV-cell interaction mechanism that was present also in unrelated cell types as bladder cancer [12] or lymphoma cells [26]. Due to the capacity of the flow cytometer to compare samples by median fluorescence intensity, for the first time in our study FLSs and ACHs were compared. The results showed that FLSs had a higher ratio of interaction with fluorescent particles with respect to ACHs. Under the paradigm of biodistribution, this result might suggest FLSs as better interactors when using ASC-EVs as therapeutic vehicles, and synovia as the preferential target tissue compared to cartilage in OA patients. Although these findings would need further confirmation, they could pave the way to a more accurate interpretation of the mechanisms of action of the so called orthobiologics, which include MSCs and their products.

Despite the importance of these results, flow cytometry cannot provide any data able to discern between adhesion of EVs on the extracellular matrix/plasma membrane and their eventual internalization, a crucial parameter to predict EV efficacy. Although with some pitfalls, confocal microscopy is commonly used to assess this parameter. In fact, confocal microscopy on 2D cultures requires very experienced operators able to discriminate between surface or internal localization of fluorescence signals. This is due to the reduced height of analyzed cells, leading also to the lack of or reduced signal on the cell surface adherent to the plastic surface [13]. On the contrary, the combination of confocal microscopy with 3D culture allows the clear detection of single cells embedded in a hydrogel-based matrix, with cell morphology resembling the in vivo condition, facilitating the recognition of fluorescent signals inside cells [27]. Here, fibrin was chosen to provide cells with a natural 3D matrix due to its ease-of-use in microfluidic applications and high biocompatibility [28]. Beyond these properties, fibrin matrix also shows some microstructural features similar to collagen [29], a major component of synovial and chondral extracellular matrix, thus being suitable to generate an in vivo-mimicking microenvironment for both synovial fibroblasts and articular chondrocytes. In our study, in both FLSs and ACHs, the results obtained in 3D cultures confirmed the flow cytometry findings on 2D cells in terms of kinetics of interaction with EVs and the superior ability of FLSs to recruit ASC-EVs. Most importantly, 3D experiments showed that only a portion of target cells were able to internalize EVs, and that at early time points FLSs again performed better than ACHs. This crucial parameter was impossible to be determined with flow cytometry, since all the cells resulted EV-positive as observed in 3D pictures when analyzing the total fluorescent signal instead of the fine-tuned localization. The distinction between efficient internalization and solely surface adhesion of EVs is of fundamental importance, making microfluidic devices compatible with hydrogel-based 3D culture a valuable tool for all those studies and future clinical tests aimed at confirming and comparing internalization of naïve or engineered EVs-therapeutic products. The future understanding of the technical or biological reasons why only a portion of cells internalized the ASC-EVs and the underlying mechanism, in the view of clinical translation, is mandatory to modify the uptake and the fusion mechanisms and thus improve this percentage. A possible technical explanation might be a partial injury of the cells during the chip preparation, although viability before fibrin embedding always resulted higher than 95%. Also, on a biological point of view, the shift from a 2D culture condition to 3D might have turned some cells to a resting state in order to adapt to the new environment, and therefore delay their intracellular metabolism and uptake kinetics. Again, the possibility to grow cells in an in vivo-like microenvironment will make microfluidics a useful tool for these approaches.

In view of ameliorating EV uptake by dissecting molecular interactions, recently it has been proposed that hyaluronic acid of the extracellular matrix may be involved in EV docking [11,30]. This may be an important way of interaction between those cells enriched in HA-based ECM, such as FLSs and ACHs [31,32], and CD44 (HA ligand) decorated EVs (as those released by ASCs and MSCs in general) [11,33]. In the orthopedic field, CD44-coated EVs were found bound to the soluble HA fibrils in both healthy and OA synovial fluids [34], suggesting HA molecules as rails for the movement of both naturally occurring or therapeutically injected EVs towards other HA-enriched structures. In this perspective, we recently demonstrated in 2D cultures that either blocking CD44 availability on ASC-EVs or removing HA from FLSs surface resulted in a reduction of EV-cell interaction [11]. These led to the hypothesis of the HA matrix as a “sponge” that increases the local concentration of EVs around the plasma membrane, thus facilitating its passive uptake or binding to specific receptors. Nevertheless, the repeal of this mechanism did not completely abolish EV interaction, suggesting the involvement of passive diffusion together with the presence of other ligand-receptor mechanisms, e.g., CD44 presence on FLSs and ACHs surface [35] together with HA coating of MSC-EVs [36]. Here, the 2D model confirmed the role of the HA matrix of EV-cell interaction for ACHs, as already shown for FLSs, suggesting a conserved mechanism of interaction [11]. The HA production was also demonstrated for both ACHs [37] and FLSs [38] in 3D systems, suggesting a conserved function, and in ACHs the expression of the major HA synthase *HAS2* was shown to be comparable between 2D cultures and 3D fibrin-based matrix similar to the one used in this report. Nevertheless, aware of possible subtle differences in HA amount between the two cell culture systems herein analyzed that do not exactly reproduce the conditions used in the mentioned reports, the 3D model clearly showed that even after the removal of the hyaluronan coating, a fluorescent signal could be detected around the cells. This reinforced the hypothesis of other molecules involved in EV docking on the cell surface. In contrast with the 2D culture, in the 3D system, after HA removal, we did not observe a diminution of the total fluorescence of cells. The difference between 2D and 3D may be due to the considerable diversity between the two experimental settings. In 2D cultures, cells are covered with a liquid phase, where EVs rapidly move by Brownian motion, and therefore the time of interaction and possibly docking with the cell surface is reduced. To increase this possibility, the HA coating is crucial for reducing EV motion by acting as flypaper and at the same time increasing their concentration along the cell surface. Conversely, in the 3D model, the fibrin gel naturally acts itself like a sponge, retaining EVs in greater number and more closely than the liquid phase in 2D culture, thus not inducing a fluorescence reduction of 4-MU treated cells. These data reinforce the hypothesis that ECM, either cell-associated or tissue-structural, plays a key role during the process of EV–cell interaction by forming a “spongy” network, embedding vesicles.

## 5. Conclusions

Future studies will be needed to confirm the role of ECM in the uptake of EVs from cells and tissues of different origins. Given the findings of our study, we suggest a combined strategy of investigation which would take advantage of both the cytofluorimetric characterization of cells grown in 2D as well as of confocal microscopy analysis on 3D cultures. In particular, hydrogel-based 3D cultures mimicking the environment/organization of soft tissues and allowing a more physiological EV-cell interaction will allow for the dissection the array of molecular mechanisms underlying EV-cell interaction. This will provide useful insights to develop new approaches aimed to improve EV docking in target cells or reduce adhesion in those cell types that should not be treated. Moreover, the combination of 2D and 3D approaches will be useful for all the studies aimed not only at the characterization of the interaction between EVs and target cells, but also at confirming the internalization of shuttled cargo.

## Supplementary Materials

The following are available online at https://www.mdpi.com/2073-4409/9/5/1180/s1, Video S1: presence of EVs around cell surface in HA-removed ACHs (left panel) after removal of the cytoplasm signal (right panel).
